# Effects of periodontal treatment on exacerbation frequency and lung function in patients with chronic periodontitis: study protocol of a 1-year randomized controlled trial

**DOI:** 10.1186/s12890-016-0340-z

**Published:** 2017-01-23

**Authors:** Sergio Romero Santos, Erika Horácio Pinto, Priscila Larcher Longo, Simone Dal Corso, Fernanda Cordoba Lanza, Rafael Stelmach, Samia Zahi Rached, Adriana Lino-dos-Santos-Franco, Marcia Pinto Alves Mayer, Sandra Kalil Bussadori, Kristianne Porta Santos Fernandes, Raquel Agnelli Mesquita-Ferrari, Anna Carolina Ratto Tempestini Horliana

**Affiliations:** 1 Universidade Nove de Julho, UNINOVE, São Paulo, Brazil; 2 Postgraduate program in Biophotonics Applied to Health Sciences, Universidade Nove de Julho, UNINOVE, Vergueiro, 235/249, CEP 01504-001 São Paulo, Brazil; 3 Postgraduate program in Rehabilitation Sciences, Universidade Nove de Julho, UNINOVE, São Paulo, Brazil; 4 Pulmonary Department, Heart Institute (InCor), School of Medicine, University of São Paulo, São Paulo, Brazil; 5 Department of Microbiology, Institute of Biomedical Sciences, University of São Paulo, São Paulo, Brazil

**Keywords:** Respiratory medicine, Dentistry, Periodontal treatment, Periodontal disease, Bronchiectasis

## Abstract

**Background:**

Chronic obstructive pulmonary disease (COPD) has been associated with periodontal disease (PD), and periodontal treatment (PT) has been connected to reduction of lung disease exacerbations. Bronchiectasis has many clinical similarities with COPD but, although it is also a chronic lung disease, to date it has not been studied with relation to PD. The aim of this study is to evaluate whether PT associated with photodynamic therapy (PDT) reduces the number of exacerbations, improves pulmonary function, periodontal clinical parameters and quality of life after 1 year of periodontal treatment follow-up.

**Methods:**

Bronchiectasis patients will undergo medical anamnesis and periodontal examination. Participants with periodontitis will be divided into two groups and PT will be performed as G1 control group (*n* = 32) – OHO (oral hygiene orientation) + supragingival treatment + simulation of using photodynamic therapy (PDT); G2 experimental (*n* = 32) – scaling and root planing + PDT + OHO. Lung function will be assessed both at baseline and after 1 year by spirometry, exacerbation history will be analyzed through clinical records monitoring. Three instruments for quality of life assessment will also be applied – *Saint George’s Respiratory Questionnaire* and Impact Profile Analysis Oral health (OHIP-14). It is expected that periodontal treatment can improve the analyzed parameters after 1 year.

**Discussion:**

Although only one study evaluates exacerbation in COPD after 1 year of PT, bronchiectasis has not been studied in the dentistry field to date. Trial registration: NCT02514226. Version #1. This study protocol receives grant from FAPESP (São Paulo Research Foundation) #2015/20535-1. First received: July 22, 2015, 1^st^ version. This protocol has been approved by the Research Ethics Committee of Nove de Julho University.

## Background

In recent years, many studies have associated periodontal disease (PD) and pulmonary diseases [1]; they have also connected decreased lung function to loss of periodontal attachment [2]. Chronic obstructive pulmonary disease (COPD) is the most studied lung disease related to PD. The pathophysiological process linking both conditions is the destruction of connective tissue and exacerbated inflammatory reaction with large recruitment of neutrophils [3]. In 1 year, periodontal treatment (PT) was able to reduce annual number of COPD exacerbations from 9 to 4 [4].

Bronchiectasis is also a chronic, irreversible lung disease characterized by permanent bronchi dilation. However, it has not yet been studied in the dental field. It is a condition that causes high morbidity, affecting patients’ quality of life [5]. In Brazil, its prevalence is estimated at 4–18% [6]. Many health professionals are not familiar with its diagnosis and, hence, it is often diagnosed as asthma or COPD due to similarities in clinical manifestations [7], such as cough and sputum production [8]. Bronchiectasis patients suffer with recurrent acute exacerbations, which sometimes require hospitalization, increasing public health expending. These patients exacerbate an average of 6.6 times per year [7] and clinical treatment is based on prevention and control of exacerbation episodes, leading to decreased number of hospitalizations.

The etiology of bronchiectasis is extremely heterogeneous and may involve genetic as well as acquired causes. The pathophysiology of bronchiectasis development involves mucociliary changes that result in bacterial colonization and subsequent chronic inflammation with damage to the bronchi [9]. Periodontal pathogens have been found in infected lung fluid [10–12]. Thus, there is biological plausibility in the assertion that a decrease in oral bacterial load could reduce the number of pulmonary exacerbations [5, 6]. To confirm this data, it is essential to measure PT contribution in improving bronchiectasis patients’ quality of life.

Hence, clinical randomized controlled trials are necessary to test if the reduction in oral infection decreases the number of exacerbations [13]. So, the hypothesis of this study is that periodontal treatment can reduce episodes of bronchiectasis exacerbation and improves bronchiectasis patients’ quality of life after 1 year. Therefore, the aim of this study is to determine if periodontal treatment leads to fewer episodes of bronchiectasis exacerbation and improves bronchiectasis patients’ quality of life after 1 year.

## Methods

A randomized, controlled, 12-month, parallel-group clinical trial, registered at www.clinicaltrial.gov (NCT02514226) will be performed. After verbal and written explanation of the study, patients will sign the Informed Consent Form approved by the Research Ethics Committee (# 1.057.901) Universidade Nove de Julho (UNINOVE). A researcher not involved in the study will enroll patients. Forty patients under medical care at InCor-FMUSP with generalized chronic periodontitis will receive periodontal treatment at Universidade Nove de Julho’s Dental Clinic, from August 2016 to September 2018.

### Sample size calculation

For this study, based on exacerbations of bronchiectasis, we assume that the difference between the groups will be one exacerbation with a standard deviation of 1.4 [14] with an effect size of 1. Thirty-two (32) patients per group will be necessary with a significance level of 5%, a power of 80% in a two-group t-test, using the statistical package Dell Statistica version 12.

### Inclusion and exclusion criteria

Patients ≥35 years, of both genders, clinically stable with more than 10 teeth and chronic moderate periodontal disease, i.e., more than 15% of examined teeth with ≥4 mm probing depth (PD) will be included [8]. Patients will be included if they have bronchiectasis, characterized clinically by chronic sputum production and dilated bronchi, and confirmed by chest computed tomography scan. Severity of bronchiectasis will be assessed according to FACED score [14].

Smokers or former smokers who have quit for less than 5 years will be excluded. Also, pregnant women, patients with other lung diseases, such as cystic fibrosis, COPD and asthma, those who are taking medications that affect periodontal condition (e.g., phenytoin or cyclosporine), patients with decompensated systemic diseases, those who required prophylactic antibiotic therapy before periodontal treatment or those who have undergone periodontal treatment less than 6 months prior will be excluded.

### Anamnesis and physical examination

In the anamnesis, questions will be asked about the patients’ general health. Demographic data (age, gender, marital status, occupation, educational level, living conditions and salary) will be collected, as well as medical history data (main complaint, current state of illness, medical and dental history, and medication use), habits and addictions (smoking, drinking, nutrition and oral hygiene).

On physical examination, clinical periodontal parameters will be collected by a single researcher. The calibration will be performed by a single researcher at six sites per tooth for probing depth (PD) and clinical attachment level (CAL) according to Pinto et al. (2016) [8]. The data will undergo the intraclass correlation coefficient test – ICC [15]. Agreement with standard examiner must be >80%.

This researcher will not be involved in the periodontal treatment of bronchiectasis patients. The following periodontal parameters will be evaluated: probing depth (PD), clinical attachment level (CAL), gingival index (GI), visible plaque index, probing bleeding (SS) and suppuration (S) (Ainamo, Bay 1975) [16].

**Fig. 1:**
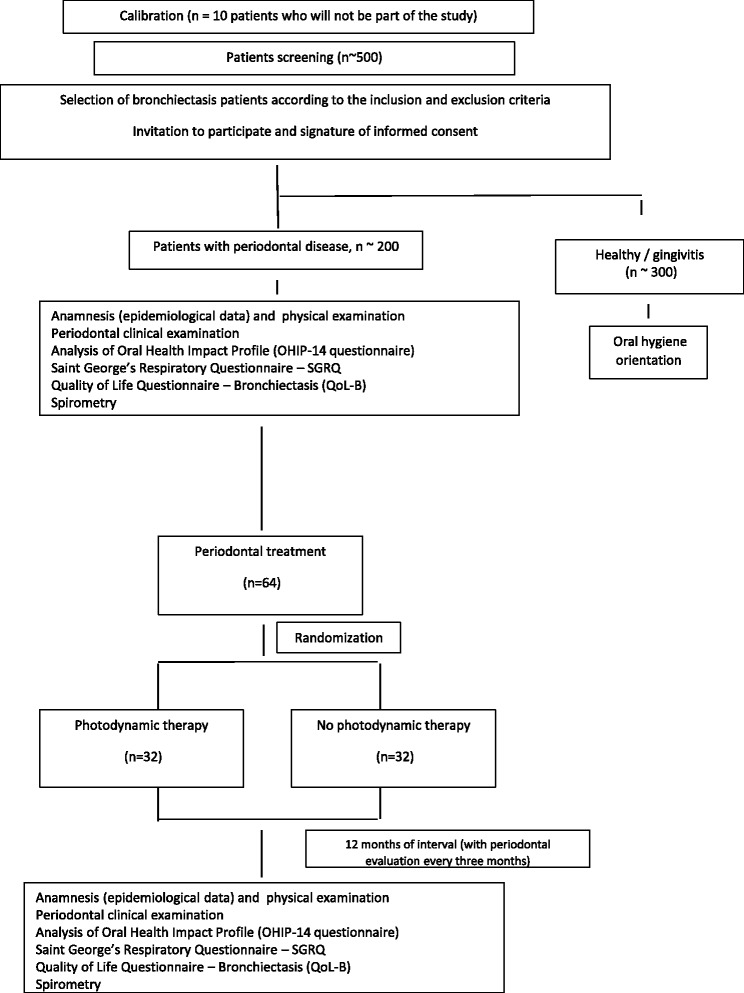
Flow chart summarizing the experimental design

Three questionnaires will be used to assess bronchiectasis patients’ quality of life: *Saint George’s Respiratory Questionnaire – SGRQ,* CoLB, and OHIP-14. They will be applied at baseline and after 12 months after periodontal treatment (Fig. 1).

### Analysis of Oral Health Impact Profile (OHIP-14 questionnaire)

The Oral Health Impact Profile (OHIP-14) is a simplified form of the original questionnaire OHIP used for assessment of oral health impact on quality of life [17]. Items are distributed among the following subscales: functional limitation, pain, psychological discomfort, physical disability, psychological disability, social disability, and deficit.

### Saint George’s Respiratory Questionnaire – SGRQ

The specific *Saint George’s Respiratory Questionnaire* (SGRQ) is used to assess quality of life in patients with pulmonary disease [18]. Responses will be evaluated by two experienced physiotherapists (S.D.C. and F.D.C.) that will be calibrated and blinded to perform the evaluation.

### Spirometry

Spirometry tests will be performed by two physiotherapists (S.D.C. and F.D.C.) using ULTIMA CPX *(MedGraphics Corporation®, St. Paul, MN, USA)* with a calibrated pneumotachograph. The technical procedures, acceptance criteria and reproducibility are recommended by the Brazilian Consensus on Spirometry [7]. All patients will perform this procedure after using a bronchodilator (salbutamol 400 μcg inhalation). Variables recorded include FVC (forced vital capacity), FEV1 (forced expiratory volume in one second timed) and FEV1/FVC. The values will be expressed in absolute values and percentage of the predicted for the Brazilian population [12].

### Randomization

Randomization will be performed as described by Pinto et al. (2016) [8] using Microsoft Excel, version 2013. Forty patients will be randomly distributed into experimental and control groups. The randomization will be blocked in groups of four patients.

Opaque envelopes will be identified with sequential numbers and inside of each of them a sheet will be inserted containing the corresponding group information, according to the random order obtained. The envelopes will be sealed and remain sealed in numerical order in a safe place until the time of periodontal treatments.

Patients will be allocated into experimental and control groups as follows:

- **Group 1** – control group (*n* = 32) – OHO (oral hygiene orientation) + supragingival treatment + simulation of using photodynamic therapy (PDT)
- **Group 2** **–** experimental (*n* = 32) – scaling and root planning + PDT + OHO (Table 1).

**Table 1:** Timeline showing the sequence of procedures and sample collection

	Study period			
	Enrolment	Allocation	Post-allocation	Close-out
Time point	-t1	0	t1	t2–12 m after periodontal treatment
Enrolment:				
Calibration of clinical examiner	x			
Informed consent	x			
Eligibility screen	x			
Allocation		x		
Interventions:				
G1–positive control group			x	
G2–experimental group			x	
Assesments				
Anamnesis (epidemilogical data) and physical examination	x			
Periodontal clinical examination	x			x
Analysis of Oral Health Impact Profile (OHIP-14 questionaire)	x			x
Saint George’s Respiratory Questionaire–SGRQ	x			x
Quality of Life Questionaire–Bronchiectasis (QoL–B)	x			x
Spirometry	x			x

### Photodynamic therapy (PDT)

Patients of Group 2 will receive photodynamic therapy. PDT associates a photosensitizer with light source in order to reduce the microbial load. The procedures will be performed as described.

- Scaling and root planning
- Application of methylene blue (0.005% - Chimiolux 10, DMC® – Purified water and methylene blue) with a carpule syringe and needle (with stop and without bevel) into the periodontal pockets >4 mm
- Wait for 3 min (Betsy [19])
- Irradiation will be performed with red laser diode (λ = 660 nm) with an output power of 100 mW (MM Optics Twin Laser, São Paulo, SP, Brazil) in all sites around a tooth (six sites per tooth) on all teeth, lastly, 1 min irradiation scanning around each tooth.
- Washing abundantly with saline until complete removal of methylene blue

Every adverse effects will be related.

### Blinding

Patients of control group will receive simulation of using photodynamic therapy (PDT).

The simulation of PDT will be performed by applying methylene blue (0.005% - Chimiolux 10, DMC® – Purified water and methylene blue) with a carpule syringe and needle (with stop and without bevel) into the periodontal pockets >4 mm. Then, wait for 3 min (Betsy [19]). Irradiation will be performed with the laser (MM Optics Twin Laser, São Paulo, SP, Brazil) turned off in all sites around a tooth (six sites per tooth) on all teeth, lastly, 1 min irradiation scanning around each tooth. Than, the operator will wash abundantly with saline until complete removal of methylene blue.

### Statistical analysis

The Liliefors test will assess the normality of the data. If the data are normal, t test (Bioestat 5.3, Pará, Brazil) will be used to compare continuous and dependent variables of Groups 1 and 2 (periodontal parameters). If the data do not have normal distribution, Mann Whitney test will be used. Baseline data will be compared with data from 12 months after treatment. The value of *p* <0.05 will be considered statistically significant. The *x* ^2^ test will be used to compare categorical variables between Groups 1 and 2. All values will be expressed as mean ± Standard Deviation.

### Study variables

The primary variable of the study will be the number of exacerbations. Secondary variables will include spirometry (lung evaluation), dental clinical parameters (periodontal examination), and quality of life parameters (SGRQ, and OHIP-14 questionnaires). These variables will be assessed at baseline and 12 months after periodontal treatment.

### Strategies for achieving adequate participant enrolment to reach target sample size

Patients will be evaluated every 3 months in order to motivate and hygiene control.

## Discussion

Lung diseases, such as chronic obstructive pulmonary disease (COPD) and bronchiectasis, cause impairment to a patient’s quality of life with large numbers of comorbidities. It has been shown that periodontal treatment (PT) and oral hygiene (OH) deceases the severity of COPD [4]; but, despite clinical and pathophysiological similarities between COPD and bronchiectasis [20], there is no study to date about the relationship of bronchiectasis with periodontal disease (PD). Patients with bronchiectasis have multiple episodes of disease exacerbations. Interventions, such as physical exercises and chronic administration of certain drugs decrease the number of such occurrences, but no intervention can achieve results within less than 6 months [21–30]. In this study, we will perform an oral intervention with a 1-year follow up. We will measure the number of exacerbations, spirometry data, dental clinical parameters and quality of life parameters (SGRQ, and OHIP-14 questionnaires). *Saint George’s Respiratory Questionnaire* is the most complete instrument, considered the gold standard for lung diseases, and OHIP-14, which analyze the impact of oral health profile. It is expected that periodontal treatment maintained for 1 year will help reduce the number of exacerbations, improve lung mechanics and quality of life in bronchiectasis patients.

## Abbreviations

COPD: Chronic obstructive pulmonary disease
FEV1: forced expiratory volume in one second timed
FVC: Forced vital capacity
OH: Oral hygiene
PD: Periodontal disease
PDT: Photodynamic therapy
PT: Periodontal treatment
SGRQ: *Saint George’s Respiratory Questionnaire*
