# Erratum to: Effects of periodontal treatment on exacerbation frequency and lung function in patients with chronic periodontitis: study protocol of a 1-year randomized controlled trial

**DOI:** 10.1186/s12890-017-0394-6

**Published:** 2017-03-14

**Authors:** Sergio dos Santos Romero, Erika Horácio Pinto, Priscila Larcher Longo, Simone Dal Corso, Fernanda Cordoba Lanza, Rafael Stelmach, Samia Zahi Rached, Adriana Lino-dos-Santos-Franco, Marcia Pinto Alves Mayer, Sandra Kalil Bussadori, Kristianne Porta Santos Fernandes, Raquel Agnelli Mesquita-Ferrari, Anna Carolina Ratto Tempestini Horliana

**Affiliations:** 1 Universidade Nove de Julho, UNINOVE, São Paulo, Brazil; 2 Postgraduate program in Biophotonics Applied to Health Sciences, Universidade Nove de Julho, UNINOVE, Vergueiro, 235/249, CEP 01504-001 São Paulo, Brazil; 3 Postgraduate program in Rehabilitation Sciences, Universidade Nove de Julho, UNINOVE, São Paulo, Brazil; 4 Pulmonary Department, Heart Institute (InCor), School of Medicine, University of São Paulo, São Paulo, Brazil; 5 Department of Microbiology, Institute of Biomedical Sciences, University of São Paulo, São Paulo, Brazil

## Erratum

The name of the first author was listed incorrectly in the original article [1]. The correct name of the first author is Sergio dos Santos Romero, and not Sergio Romero Santos. The correct citation of his name is “Romero SS” not “Santos RS”.
